# Suicide and suicide attempts in people with severe mental disorders in Butajira, Ethiopia: 10 year follow-up of a population-based cohort

**DOI:** 10.1186/1471-244X-14-150

**Published:** 2014-05-23

**Authors:** Teshome Shibre, Charlotte Hanlon, Girmay Medhin, Atalay Alem, Derege Kebede, Solomon Teferra, Gunnar Kullgren, Lars Jacobsson, Abebaw Fekadu

**Affiliations:** 1 Department of Psychiatry, Addis Ababa University, College of Health Sciences, School of Medicine, Addis Ababa, Ethiopia; 2 Ontario Shores Centre for Mental Health Sciences, University of Toronto, Toronto, Canada; 3 Health Services and Population Research Department, King’s College London, Institute of Psychiatry, Centre for Global Mental Health, London, UK; 4 Aklilu Lemma Institute of Pathobiology, Addis Ababa University, Addis Ababa, Ethiopia; 5 College of Health Sciences, School of Public Health, Addis Ababa University, Addis Ababa, Ethiopia; 6 WHO Regional Office for Africa, Brazzaville, Republic of Congo; 7 Department of Clinical Sciences, Division of Psychiatry, Umeå University, Umea, Sweden; 8 King’s College London, Institute of Psychiatry, Department of Psychological Medicine, Centre for Affective Disorders and Affective Disorders Research Group, London, UK; 9 College of Health Sciences, School of Medicine, Department of Psychiatry, Addis Ababa University, PO Box 9086, Addis Ababa, Ethiopia

**Keywords:** Suicide, Schizophrenia, Major depression, Bipolar I disorder, Developing countries, sub-Saharan Africa, Ethiopia

## Abstract

**Background:**

People with severe mental disorders (SMD) are at higher risk of suicide. However, research into suicide attempts and completed suicide in people with SMD in low- and middle-income countries is mostly limited to patients attending psychiatric facilities where selection bias is likely to be high.

**Methods:**

A population-based cohort of 919 people with SMD from rural Ethiopia (who received standardized clinician diagnoses of schizophrenia (n = 358) major depressive disorder (n = 216) and bipolar I disorder (n = 345)) were followed up annually for an average of 10 years. The Longitudinal Interval Follow-up Evaluation chart was administered by psychiatrists and used to evaluate systematically suicidal behavior and risk factors, which may be amenable to intervention.

**Results:**

Over the follow-up period, the cumulative risk of suicide attempt was 26.3% for major depression, 23.8% for bipolar I disorder and 13.1% for schizophrenia, (p < 0.001). The overall incidence of completed suicide was 200.2/100,000 person-years (CI = 120.6, 312.5). Hanging was the most frequent method used (71.5%) for both attempters and completers. Most people who completed suicide were successful on the first attempt (84.2%), but the case-fatality rate for suicide attempt was 9.7%. In the adjusted logistic regression model, being currently married (Adjusted OR) =2.17, 95% CI = 1.21, 3.91), and having a diagnosis of bipolar I disorder (Adjusted OR = 2.59, 95% CI = 1.57, 4.26) or major depression (Adjusted OR = 2.71, 95% CI = 1.60, 4.58) were associated significantly with increased risk of suicide attempts.

**Conclusion:**

In this sample of people with SMD from a rural setting, the rate of suicide was high. Initiatives to integrate mental health service into primary care need to focus on limiting access to suicide methods in people with SMD in addition to expanding access to mental health care.

## Background

Suicidal behavior is a major contributor to morbidity and mortality worldwide. Over 760,000 (85%) suicides occur in low and middle-income countries (LMICs) [1]. Over 90% of all suicides are believed to occur in the context of mental disorders [2-7] mostly mood disorders, and alcohol and substance use disorders [7,8]. Severe mental disorders (SMDs), including schizophrenia, bipolar disorder and psychotic depression), are associated with increased risk of suicide and suicide attempts compared to the general population [7,9,10]. Indeed, in schizophrenia, suicide is the commonest cause of premature death [11,12]. Even though SMDs have a relatively low prevalence (1-2%), a recent review estimated that up to 12 percent of all deaths due to suicide are attributable to schizophrenia [13]. People with schizophrenia are more likely to use serious and violent methods in response to hallucinatory voices and delusions compared to patients with major depression [14].

Suicide might be a hidden cause of death in traditional societies because of the high levels of stigma and associated religious and cultural condemnation [15,16]. However, most research on suicide has been carried out in the more affluent countries [9,17,18]. Furthermore, most studies from traditional societies focus on suicide in the general population [6,19], and there is extremely limited information on the epidemiology of fatal and non-fatal suicidal behavior among people with SMD [20,21]. Factors contributing to this situation include poorly developed and under-resourced systems to collect and report data on suicide, coupled with religious and cultural sensitivities that render reporting unacceptable [15,16]. The few existing reports of suicide and suicide attempts in people with SMD in LMICs are restricted to patients attending psychiatric facilities, where selection bias is high due to barriers to help seeking and the large treatment gap [22,23].

In studies conducted in Ethiopia, the lifetime prevalence of attempted suicide was found to be 3.2% for a rural adult population in Butajira [15], 0.9% for an urban community in Addis Ababa [24], 14.3% for a high school student population [25] and 19.2% for a psychiatric out-patient clinic attendants [23]. Earlier hospital-based studies from Ethiopia, reported higher rates of suicide attempts among younger age groups [26,27]. In a systematic study of police and hospital records from Addis Ababa, the rate of completed suicide was found to be 7.76/100,000 persons per year [27], with higher rates of attempts in males. . There are no studies from Ethiopia looking at suicidal behavior in people with SMD.

In this paper, we report on suicide attempts, completed suicide and associated factors based on prospective data gathered from a large, population-based cohort of people with SMD in rural Ethiopia, who have been under continuous follow-up for over 10 years. The objectives were to quantify suicidal behaviors and identify risk factors that may be amenable to intervention. We hypothesized that the rate of suicide attempts and completed suicide would be lower than that found in high income countries due to strong social cohesion and support systems.

## Methods

### Setting

This study is part of the Butajira Study on Course and Outcome of Severe Mental Disorders in Ethiopia, which has been described in detail previously [28]. The Butajira district is located about 135 km south of Addis Ababa, the capital city of Ethiopia. In keeping with the general improvement in infrastructure and health service coverage across the whole country over the past 20 years, access to health care has expanded markedly in rural Butajira. There is now one zonal referral hospital, 11 health centers and over 40 health posts, each staffed by two community-based health extension workers. Except for one outreach site run by this research project, psychiatric care is only rendered at the Butajira referral hospital. Two psychiatric nurses run this service. Most people in the area use traditional methods for treating SMDs and those who seek modern treatment usually continue to use these traditional methods in addition to modern care [29]. The main livelihood for people in the district is farming, with the cash crops of khat (an amphetamine-like stimulant) and chilli peppers grown in the highland and lowland areas, respectively.

### Study design

A population-based cohort study.

### Initial recruitment

Initial recruitment of the Butajira cohort occurred between March 1998 and May 2001. Two-stage screening was carried out in the total adult population of the district aged between 15 and 49 years (n = 68,378; 83.0% of eligible population) in order to identify people with schizophrenia, bipolar I disorder and major depression (MDD) [28,30]. The psychosis and mood disorder sections of the Composite International Diagnostic Interview [31] (CIDI) 2.1 were supplemented by the Key Informant method in order to find possible cases [30]. This was then followed by a diagnostic clinician interview using the Amharic Version of the Schedules for Clinical Assessment in Neuropsychiatry (SCAN 2.1) [32]. Additional incident cases were also identified over the ensuing two years. Patients in this study were followed up monthly for a period of between 10 to 13 years.

### Study population

A total of 919 patients; 358 patients with the diagnosis of schizophrenia, 216 with MDD and 345 with bipolar I disorder were included in the study. Seventy-five of these patients were enrolled in the study as incident cases; schizophrenia (n = 40), MDD, (n = 8) and bipolar I disorder (n = 27) over two years after the initial survey was completed. Although less than 10% of the cohort had ever used any modern psychiatric service before recruitment, all patients were given free access to basic psychiatric treatment throughout the follow-up period. Drug treatment included first generation oral and depot antipsychotic medications and tricyclic antidepressants. An outreach service station was also initiated by the study project so that patients living in distant villages could access the service on scheduled dates. The psychiatric service team included lay project outreach workers who made monthly home visits, facility-based psychiatric nurses and a medical doctor. Psychiatrists provided telephone consultation or assessed patients in the Butajira clinic as needed.

### Inclusion and exclusion criteria

All patients with established DSM-IV/ICD-10 diagnoses of schizophrenia, MDD and bipolar I disorder and who had at least one follow-up assessment after enrollment were included in the analysis in this report. Suicidal gesture or attempt was defined as a self-inflicted act associated with intent to die or use of a method with potential for lethality.

### Follow-up

Patients were followed up for a period varying from one month to 13 years, with a mean follow-up period of 10 years. Patients who needed treatment were scheduled to visit the psychiatric clinic monthly. On each monthly visit the presenting symptoms, examination findings and prescribed treatments were recorded. Annual research assessments were carried out by psychiatric nurses and medical doctors, including systematic assessment of symptom profile, diagnosis, treatment and adverse effects of drugs, changes in work and living arrangements, Global Assessment of Function (GAF) [33] and Family Interview Schedule (FIS) [32].

For the final follow-up assessment, trained psychiatrists carried out a clinical assessment and completed the Longitudinal Interval Follow-up Evaluation (LIFE) [34] chart for each person in the cohort. The LIFE-Chart data were generated from a range of sources, including data from patients, family members, the baseline and annual follow-up data, monthly clinic visit notes, the project outreach workers who made monthly home visits and the psychiatric nurses who had treated the patients throughout the follow-up period. The LIFE-Chart required the psychiatrists to make retrospective month-by-month ratings of psychopathology, medication treatment, substance use and psychosocial functioning. A narrative account summarized the individual patient data over the periods of the follow-up. Each patient’s baseline and follow-up data were summarized before contacting the patient. In situations where patients could not come to the psychiatric clinic for the final assessment, psychiatrists made home visits to do the assessments. Data on the timing of completed suicide was collected continuously during follow-up of the cohort, with verification and additional information on methods of suicide obtained at the final LIFE-chart assessment. Verbal autopsy documents that were completed within four weeks of report of a patients’ death to determine the possible immediate cause of death were also included in the review for the deceased cases.

Consultant psychiatrists (AF and TS) supervised the ratings. One of the psychiatrists (AF) was trained in LIFE- Chart in a specialized center in the USA and trained the psychiatrists who completed LIFE Chart.

### Data analysis

Completed LIFE-Chart questionnaires were cross-checked for completeness on a daily basis. The Statistical Program for Social Science (SPSS version 15) was used to computerize the data. Data were then transferred to STATA (version 11) for analysis. Since the amount of missing data was under 2%, complete case analysis was carried out. Sociodemographic (age, sex, marital status, areas of residence, religion, education and having children) and clinical factors (diagnosis, percent time on psychotropic medication, history of alcohol and khat abuse or dependence, level of functioning at enrollment measured with the Global Assessment of Functioning) known to be associated with suicidal behavior were included in a multiple logistic regression model. Multivariable analysis of factors associated with completed suicide was not carried out because of the small number of completed suicide during the follow-up period. Poisson regression was used to model the incidence of suicide attempt taking number of attempts as an outcome and duration of follow-up as an offset variable.

### Ethical considerations

Ethical approval was obtained from Addis Ababa University, Faculty of Medicine. Study participants provided consent to participate in the study. Free psychiatric consultation and medication were provided for patients through the study project.

## Results

LIFE chart were completed for a total of 919 participants. Face to face interviews were carried out by psychiatrists on 68.2% (n = 627) of the participants. Information for 31.8% (n = 292) of participants relied on data extracted from follow-up documents, informants who knew the patient very well and project field staff. Of these 292 patents, 39.7% (n = 116) were still under active follow-up but could not attend for the LIFE assessment, 40.7% (n = 119) were deceased by the time of the LIFE interview, 16.4% (n = 48) had migrated out of the study site and hence were not traceable and 3.0% (n = 9) were vagrants and could not be located. The LIFE chart evaluation used clinical and research documents, which were available for all 919 and face to face interview by psychiatrists carried out to complete the LIFE chart on 66.5% (n = 627) of the participants. The face to face assessment and document reviews were supplemented with collateral information whenever required.

### Suicidal behavior

The prevalence of at least one suicide attempt during the 10-year period was 20.2% (n = 186), with the number of attempts ranging from 1 to 5. The ratio of suicide attempters to completers was 10.3:1. The lifetime prevalence of suicide attempt was 26.5% for patients with MDD, 23.8% for patients with bipolar I disorder and 13.1% for patients with schizophrenia. The overall rate of suicide attempt was significantly higher among those with mood disorders (bipolar disorder and major depression) compared to those with schizophrenia (x^2^(1) = 12.5; p < 0.001). The prevalence of suicide attempts stratified by patients’ baseline and follow-up characteristics is summarized in Tables 1 and 2. The prevalence of suicide attempt was higher in women (23.6% vs. 18.2%; p = 0.046), those who were married (p < 0.001) and those who did not have a child (p = 0.001).

**Table 1:** Baseline and follow-up characteristics of people with severe mental disorders who attempted or completed suicide over the 10-year follow-up period (categorical variables)

**Characteristics**	**Total sample (n = 919)**	**Exhibited suicidal behavior (n = 186)**		
	**N**	**N**	**%**	**p-value**
*Baseline characteristics*				
**Gender**				
Male	572	104	18.2	0.046
Female	347	82	23.6	
**Age group at enrollment (years)**				
<20	92	18	19.6	0.577
20-29	349	65	18.6	
30-39	306	70	22.9	
>40	160	31	19.4	
**Marital status**				
Never married	347	47	13.5	<0.001
Married	449	116	25.8	
Widowed, divorced or separated	109	21	19.3	
**Child status**				
None	479	118	24.6	0.001
1 or more child	440	68	15.5	
**Educational status**				
Non-literate	573	124	21.6	0.206
Literate	331	60	18.1	
**Employment**				
Unemployed	429	80	18.7	0.261
Employed	490	106	21.6	
**Religion**				
Muslim	644	121	18.8	0.212
Orthodox Christian	222	52	23.4	
Protestant Christian	51	13	25.5	
**Area of residence**				
Urban	187	34	18.2	0.433
Rural	732	152	20.8	
**Speed of illness onset**				
Acute (within 3-months)	638	143	22.4	0.133
Insidious (3-12 months)	83	15	18.1	
Insidious (>12 months)	82	11	13.4	
**Diagnosis**				
Schizophrenia	358	47	13.1	<0.001
Bipolar I Disorder	345	82	23.8	
Major Depressive Disorder	216	57	26.3	
*Follow-up characteristics*				
**% of follow-up receiving psychotropic medication**				
≥50% of follow-up period	198	42	21.2	0.701
<50% of follow-up period	721	144	20.0	
**Percentage of time adherent to medication**				
<25%	182	43	23.6	0.348
25-49%	106	27	25.5	
50-75%	156	29	18.6	
>75%	338	63	18.6	
Not prescribed	136	24	17.7	
**History of alcohol/khat abuse or dependence**				
No	750	148	19.7	0.421
Yes	169	38	22.5	
**Level of social functional impairment on last assessment**				
No impairment	208	42	20.2	0.094
Mild impairment	97	25	25.8	
Moderate	174	43	24.7	
Severe	440	76	17.3	

**Table 2:** Baseline and follow-up characteristics of people with severe mental disorders who attempted or completed suicide over the 10-year follow-up period (continuous variables)

	**Non-attempters**		**Attempters and completers**		
	**Number**	**Mean (SE)**	**Number**	**Mean (SE)**	**P-value**
*Baseline characteristics*					
**Enrollment global assessment of functioning score**	733	45.5 (0.81)	186	45.3 (0.79)	0.919
**Age at first clear recognition of symptoms (years)**	706	22.9 (0.34)	179	22.3 (0.32)	0.460
**Carer burden**	677	1.2 (0.03)	168	1.1 (0.04)	0.461
**Carer stigma experience score**	676	0.6 (0.2)	168	0.5 (0.2)	0.328
*Follow-up characteristics*					
**Schizophrenia**					
**Mean % time in episode**	312	28.7 (1.88)	47	26.4 (1.80)	0.654
**Mean % time in remission**	312	30.3 (2.00)	47	34.8 (2.04)	0.423
**Bipolar I**					
**Mean % time in episode**	263	3.0 (0.50)	82	3.3 (0.46)	0.762
**Mean % time in remission**	263	78.3 (2.08)	82	78.4 (1.92)	0.982
**Depression**					
**Mean % time in episode**	158	6.6 (1.40)	57	8.1 (1.23)	0.565
**Mean % time in remission**	158	80.3 (2.10)	57	74.1 (2.47)	0.147

There was no significant age difference between people with and without suicidal behavior (see Tables 1 and 2). There was no significant association between suicidal behavior and any of the following variables: family experience of stigma, caregiver burden, alcohol or khat abuse during the follow-up period, history of poor treatment adherence or percentage of the follow-up time in episode of mental disorder or in remission (Tables 1 and 2).

Results from a multivariable logistic regression model are summarized in Table 3. While the significant univariate association between female gender and suicidal behavior disappeared in the multivariable model (OR = 1.5, 95% CI = 0.69, 1.59) other factors were significantly associated with the odds of suicidal behavior. Being at least 40 years of age at enrollment was inversely associated with odds of suicide attempts (OR = 0.41, CI = 0.18, 0.93). Compared to people who had never married, those who were married had an increased odds of having one or more suicide attempts (OR = 2.17, CI = 1.21, 3.91). Compared to patients with the diagnosis of schizophrenia, patients with the diagnosis of bipolar I disorder (OR = 2.59, CI = 1.57, 4.26) and those with the diagnosis of MDD (OR = 2.71, CI = 1.60, 4.58) had an increased odds of suicide attempts. Having a history of alcohol or khat abuse was also associated significantly with increased odds of suicide attempts (OR = 1.84, CI = 1.14, 2.96). Higher baseline GAF scores were associated with lower odds of suicide attempts (OR = 0.99, CI = 0.98, 0.99). Compared to those who only attempted suicide, those who successfully committed suicide were likely to be men, to receive a diagnosis of schizophrenia and to be on treatment for longer (Table 4). In relation to incidence of suicide attempt, increased incidence rate was associated with marriage and loss of marriage, having a diagnosis of mood disorders (both bipolar and major depressive disorder), history of substance abuse, receipt of psychotropic medications for a prolonged period and illness severity at enrollment (Table 5). The interaction of baseline severity of illness and treatment status was not significant; however the inclusion of the interaction term resulted in non-significance of the effect of treatment duration on the incidence of suicide attempt.

**Table 3:** Univariate and multivariable analysis of factors associated with suicidal behavior (attempted or completed suicide) during the course of follow-up among the Butajira cohort

**Characteristics**	**Crude OR**	**95% CI**	**p-value**	**Adjusted OR**	**95% CI**	**p-value**
**Gender**						
** Female**	Ref.			Ref.		
** Male**	0.71	0.52,0.99	0.047	1.05	0.69,1.59	0.825
**Age group at enrolment (years)**						
** <20**	Ref.			Ref.		
** 20-29**	0.94	0.53,1.68	0.837	0.66	0.35,1.26	0.210
** 30-39**	1.22	0.68,2.18	0.503	0.65	0.32,1.31	0.228
** ≥40**	0.99	0.51,1.89	0.971	0.41	0.18,0.93	0.033
**Marital status**						
** Never married**	Ref.			Ref.		
** Married**	2.22	1.53,3.23	<0.001	2.17	1.21,3.91	0.010
** Separated/divorce/widowed**	1.52	0.86,2.68	0.146	1.69	0.86,3.33	0.130
**Having children**						
** No**	Ref.			Ref.		
** Yes**	1.79	1.28,2.49	0.001	1.20	0.70,2.01	0.508
**Educational status**						
** Non-literate**	Ref.			Ref.		
** Literate**	0.80	0.57,1.13	0.207	0.85	0.57,1.28	0.441
**Religion**						
** Muslim**	Ref.			Ref.		
** Christian**	0.76	0.52,1.09	0.137	0.69	0.46,1.03	
** Protestant**	1.12	0.55,2.26	0.755	1.21	0.58,2.54	0.607
**Area of residence**						
** Urban**	Ref.			Ref.		
** Rural**	1.18	0.78,1.78	0.433	1.01	0.64,1.60	0.957
**Diagnosis**						
** Schizophrenia**	Ref.			Ref.		
** Bipolar I**	2.07	1.40,3.07	<0.001	2.59	1.57,4.26	<0.001
** MDD**	2.39	1.56,3.69	<0.001	2.71	1.60,4.58	<0.001
**History of alcohol/khat abuse**						
** Absent**	Ref.			Ref.		
** Present**	1.18	0.79,1.77	0.422	1.84	1.14-2.96	0.013
**Follow-up treatment status**						
** Received treatment for < 50% of the follow-up period**	Ref.			Ref.		
** Received treatment for > 50% of the follow-up period**	1.08	0.73,1.59	0.701	1.45	0.95,2.24	0.088
**Global assessment of functioning score at enrollment**	1.00	0.99,1.01	0.918	0.99	0.98, 0.997	0.009

**Table 4:** Factors associated with fatal outcome in suicide attempters

**Characteristics**	**Non-fatal outcome**	**Fatal outcome**	**p-value**
	**Number (%)**	**Number (%)**	
*Baseline demographics*			
**Gender**			
Male	88 (83.8)	17 (16.2)	0.002
Female	80 (97.6)	2 (2.4)	
**Age group at enrolment**			
<20	17 (89.5)	2 (10.5)	0.590
20-29	58 (89.2)	7 (10.8)	
30-39	65 (92.9)	5 (7.1)	
>40	26 (83.9)	5 (16.1)	
**Marital status**			
Never married	42 (87.5)	6 (12.5)	0.840
Married	105 (90.5)	11 (9.5)	
Others	19 (90.5)	19 (10.3)	
**Religious affiliation**			
Orthodox Christian	45 (86.5)	7 (13.5)	0.349
Muslim	110 (90.2)	12 (9.8)	
Protestant	13 (100)	0 (0)	
**Area of residence**			
Urban	31 (91.2)	3 (8.8)	0.775
Rural	137 (89.5)	16 (10.5)	
**Child status**			
No children	61 (88.4)	8 (11.6)	0.620
At least one child	107 (90.7)	11 (9.3)	
*Caregiver experience at baseline*			
**Stigma score**	Mean =1.09	Mean =1.28	0.452
	SE = 0.08	SE = 0.30	
**Burden score**	Mean =0.54	Mean =0.43	0.396
	SE = 0.04	SE = 0.12	
*Clinical features at baseline*			
**Diagnosis**			
Schizophrenia	39 (81.3)	9 (18.8)	0.036
Bipolar-I Disorder	74 (90.2)	8 (9.8)	
Major Depressive Disorder	55 (96.5)	2 (3.5)	
**History of suicidal thoughts at enrolment**			
Absent	97 (90.7)	10 (9.4)	0.943
Present	56 (90.3)	6 (9.7)	
**History of suicidal attempts at enrolment**			
Absent	129 (91.5)	12 (8.5)	0.133
Present	23 (82.1)	5 (17.9)	
**GAF score at baseline**	Mean = 45.5	Mean = 45.3,	0.919
	SE = 0.82	SE = 1.6	
**Age at first clear recognition of symptoms**	Mean =22.2	Mean = 23.6	0.499
	SE = 0.7	SE = 1.4	
*Clinical features during follow-up*			
**Schizophrenia illness course (n = 312)**			
% time in psychotic episode	Mean =25.8	Mean =37.5	0.344
	SE = 5.0	SE = 13.8	
%time in remission	Mean =24.4	Mean =32.2	0.871
	SE = 5.9	SE = 11.5	
**Bipolar I disorder illness course (n = 263)**			
% time in manic or depressive episode	Mean =3.2	Mean =4.2	0.719
	SE = 0.9	SE = 2.8	
% time in remission	Mean =78.3	Mean =79.9	0.892
	SE = 3.6	SE = 11.9	
**Major depressive disorder (n = 158)**			
% time in depressive episode	Mean =8.0	Mean =10.5	0.829
	SE = 2.1	SE = 7.3	
% time in remission	Mean =73.5	Mean =90.2	0.460
	SE = 4.2	SE = 9.8	
**Follow-up treatment status**			
Received treatment for > 50% of the follow-up period	33 (78.6)	9 (21.4)	0.006
Received treatment for < 50% of the follow-up period	135 (93.1)	10 (6.9)	
**Alcohol/khat abuse/dependence**			
No	136 (91.3)	139 (8.7)	0.198
Yes	32 (84.2)	6 (15.8)	

**Table 5:** Incidence of suicide attempt and associated factors

**Characteristics**	**IRR**	**95% CI**	**p-value**
**Gender**			
Female	Ref.		
Male	0.99	0.74,1.31	0.920
**Age group at enrolment (years)**			
<20	Ref.		
20-29	0.77	0.49,1.23	0.281
30-39	0.91	0.56, 1.50	0.719
≥40	0.90	0.52, 1.56	0.713
**Marital status**			
Never married	Ref.		
Married	2.08	1.39,3.10	<0.001
Separated/divorce/widowed	1.83	1.16,2.88	0.009
**Having children**			
No	Ref.		
Yes	0.88	.63, 1.25	0.491
**Educational status**			
Non-literate	Ref.		
Literate	0.87	0.66,1.15	0.337
**Religion**			
Muslim	Ref.		
Orthodox Christian	0.76	0.58,1.00	0.048
Protestant Christian	0.97	0.59,1.62	0.917
**Area of residence**			
Urban	Ref.		
Rural	0.99	0.73,1.35	0.960
**Diagnosis**			
Schizophrenia	Ref.		
Bipolar I	1.51	1.07,2.11	<0.018
Major Depressive Disorder	1.84	1.30,2.61	0.001
**History of alcohol/khat abuse**			
Absent	Ref.		
Present	1.51	1.10,2.08	0.012
**Percentage of follow-up time on treatment**			
For < 50% of the follow-up time	Ref.		
For > 50% of the follow-up time	1.38	1.03,1.84	0.030
**GAF categories at baseline**			
60 and above	Ref		
<60	1.37	1.03,1.81	0.029

### Completed suicide

**Figure 1:**
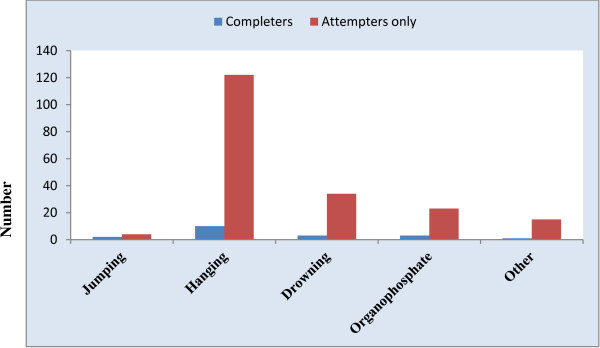
Mode of suicide attempt in completers and attempters only (Other: includes medication overdose (1 patient who was a completer), holding live electricity wires and cables, running into moving vehicles and stabbing).

Hanging was the most frequent method used by both attempters and completers groups (71.5%), followed by drowning (19.9%) and organophosphate poisoning (14%). Twenty-nine patients (15.6%) used multiple methods in an attempt to end their life. Patients with a diagnosis of bipolar I disorder or schizophrenia used more aggressive methods; jumping from a height, hanging, and drowning by falling into narrow water wells (Figure 1). There was no apparent difference in method between those who died and those who survived from the attempts.

Male gender was associated significantly with suicide attempt that resulted in a fatal outcome [16.2% (n = 17) in males compared with 2.4% (n = 2) in females; p = 0.002)]. Of suicide attempts, the percentage resulting in death was significantly lower in patients with diagnoses of MDD or bipolar I disorder compared to patients with schizophrenia: 18.8% (n = 9) in schizophrenia, 9.9% (n = 8) in bipolar I disorder and 3.5% (n = 2) in MDD; (p-value = 0.036). Over the10-years of follow-up, the percentage of patients committing suicide was 2.5% for schizophrenia, 2.3% for bipolar I disorder and 0.92% for MDD. The incidence of death from suicide was highest for patients with a diagnosis of schizophrenia (253.7/100,000 person years of follow-up, CI = 116.1, 481.0), followed by bipolar I disorder (216.5, 95% CI = 93.5, 426.1) and MDD (89.1, 95% CI = 10.8, 321.4). The overall incidence of completed suicide in the cohort was 200.2/100,000 person-years (CI = 120.6, 312.5).

Suicide attempts were increased in patients who had received recommended treatment for ≤50% of their follow-up time (93.1%; n = 135) compared to those who received recommended treatment for more than 50% of their follow-up time. Fatal outcome of a suicide attempt was increased in patients who had received treatment for more than 50% of their follow-up time compared to those who received treatment for ≤50% of the follow-up time [21.4% (n = 9) versus 6.9% (n = 10); p = 0.006]. Almost all patients in the fatal outcome group (n = 17; 90.0%) had symptoms suggestive of acute illness in the week preceding their death, while only 52.6% (n = 10) were taking their medications regularly around the time when death occurred.

## Discussion

The incidence of completed suicide and suicide attempts in this large, population-based, clinician-diagnosed cohort of people with SMD in rural Ethiopia was comparable to that seen in people with SMD in high-income country settings [2,18]. Despite the potential under-reporting of suicide behavior in many societies [15,35], suicide is a global public health problem and mental illness is known to be the strongest risk factor [36]. In this study, suicide attempts were more commonly seen in people with mood disorders, especially MDD, compared to those with schizophrenia; however, amongst suicide attempters, completed suicide was substantially higher in people with schizophrenia. This finding is in line with other follow-up studies that reported significantly higher rates of suicide attempt among patients with depression [37].

The 10 year prevalence of suicide attempts in patients with major depression (26.5%) found in this study is higher than the lifetime prevalence of around 16% in people with MDD reported in clinic-based studies in some high-income countries [38]. However, the finding on bipolar disorder is comparable with other reports where up to 29% of patients with bipolar disorder are estimated to attempt suicide at least once in their life-time [39], with one study reporting a five year prevalence of 18% [40]. A European longitudinal study of bipolar disorder patients reported lifetime prevalence of suicide attempt of 29.9%, which is comparable to this study finding and that the risk for suicide attempt increases with increased duration of illness [17]. Although under-reporting is difficult to avoid, the use of diverse sources of information to collect the data and the close monitoring over the follow-up period in our study might have mitigated the impact of under-reporting. In an earlier community-based study from the same area of Ethiopia, the lifetime prevalence of suicide attempt was reported to be 14.6% in people with minor depressive disorder [41]. In our study, greater severity of illness is likely to explain the difference in suicidal behavior and lends indirect validity to the construct of depression in this setting. Diagnosed MDD in isolation, or when co-morbid with another axis I disorder, is a well-established major risk factor for suicidal behavior worldwide [17,38,42].

The rate of suicide attempts in this study was also much higher than the lifetime prevalence of 6.9% that was reported from the same population at baseline [43]. Longer duration of illness might explain this difference.

Suicide is common among patients with schizophrenia and it is estimated that about 10% of patients with schizophrenia will eventually commit suicide and over four times that number make attempts [44]. Actual reports of prevalence of suicide attempts vary from 19.8% [20] to 34.5% [45]. Our finding of suicide attempt in 13.1% of patients with the diagnosis of schizophrenia is less than that reported from affluent countries. Most suicidal behavior and suicide-related deaths in people with schizophrenia occur in the early phase of the illness, with the risk of death from suicide estimated to be around 5 or 6% [46,47]. People with schizophrenia in our cohort had a mean duration of illness of over 10 years at enrolment, which might have contributed to low rates of suicidal behavior during the follow-up period. The overall ratio of attempted to completed suicide in our study was at the lower end of what is reported elsewhere for general population studies (8-25:1) [48]. This was an unexpected finding given the relative lethality of methods used for suicide attempts in this setting.

Increased incidence of suicide attempt and a fatal outcome from a suicide attempt was more likely among patients who received treatment with psychotropic medication for more than half of the follow-up time. There could be a number of possible explanations for the apparently non-beneficial effect of medication on suicidal behavior in our study. First, the evidence for beneficial effects of psychotropic medication on reducing suicide attempts and suicide is largely restricted to antidepressant medication in people with MDD [38] and mood-stabilizers, particularly Lithium [49], in people with bipolar disorder [50]. Second, although treatment with antipsychotic medication improves the quality of life and reduces symptoms in patients with schizophrenia, evidence for association between treatment with first generation antipsychotic medications and lowering of suicide risk is inconclusive [13]. This is also true for other antipsychotic medications, except for clozapine, which is approved by the FDA for treatment of suicidal behavior in schizophrenia and schizoaffective patients [51]. In this study, most completed suicides (47.4%) occurred in people with schizophrenia. Furthermore, mood stabilizer medication was not available for the vast majority of patients; instead, people with bipolar disorder were treated with a combination of first generation antipsychotic medication and antidepressant medications. Perhaps the most likely explanation for the finding is, however, reverse causality. People with severe clinical symptoms are more likely to be prescribed psychotropic medication and to receive medication for longer periods of time. For example, higher level of functional impairment and symptomatology measured with the GAF at baseline was associated with an increased incidence of suicide attempts (Table 5).

The burden from suicide, due to loss of potential years of life, and the associated economic and societal consequences are more pronounced for younger age groups. This has been shown in many general population studies [19,52,53]. Among people with SMD in our study, we observed no significant difference in the mean age of suicide attempters compared to those who never attempted suicide, and between attempters and completers. Those who completed suicide were distributed across all age groups in this population, possibly because having a psychiatric disorder itself is a risk factor, independent of age and other factors [37,54].

Despite the widely held belief that people who are married and have a more stable life have lower rates of suicide attempts and completed suicide [44,55,56], there are controversies around this finding. While increased suicide risk has been reported for separated, divorced and widowed populations in other studies of suicide in people with SMD in LMICs [13,20], earlier Ethiopian study conducted in the capital city did not find any association between suicide attempt with marital status [24]. In a general population study from India, being separated, divorced or widowed was protective against suicide in women [19]. A case-control study from Pakistan also found marriage to increase the risk of suicide [57]. The finding that married people had significantly more suicide attempts in this study after adjusting for confounders might be a real reflection of the stressful life situation in LMICs for people who are married, independent of age and gender. An earlier study from the same area reported intimate partner violence to be a risk factor for suicide attempt [58]. We did not have data on domestic violence to look into the association between suicide attempt and domestic violence among married women.

The findings that male gender, young age and previous history of suicidal attempt were associated with increased risk of suicide accorded with established findings across schizophrenia studies [13,42,44,45,47]. Past history of suicidal attempt is also reported to be a risk factor for suicidal behavior in mood disorder patients as well [59], but did not show any significant association in our study, which might be because of the small number of fatal outcomes in our study population.

In contrast to other studies, less than 20.0% of our study population had a history of alcohol or other substance abuse. Furthermore, most substance misuse was limited to chewing khat leaves (amphetamine like substance), which is grown widely in the area. The association between history of alcohol and substance abuse and suicidal behavior is a well-established finding in general population studies and psychiatric patients with different diagnosis [9,17,42,45,60] and our finding is in line with these studies.

The finding that suicide attempt was a more frequent phenomenon among female patients and completed suicide was significantly more common among male patients in our study also concurs with other studies [8,17,19,39].

Violent methods of attempt seem to be more important than the number of attempts in our population as 16/19 (84.2%) of the patients with completed suicide did so at the first attempt where almost all male patients used aggressive methods such as jumping from a height, hanging and drowning. The finding that hanging is the commonest method used is in agreement with other studies in Ethiopia [23,24] and in the Butajira area [15]. Methods such as self poisoning with organophosphate chemicals and drowning are also commonly reported and have a potential for prevention. With the government’s policy of encouraging farmers to increase their yields using pesticides and fertilizers, there might be increased access to such chemicals in remote rural areas, increasing the risk of death from attempted suicide.

Prevention of access to common methods of suicide, especially restricting access to pesticides, and providing treatment for depression and alcohol use disorders has been recommended by earlier researchers from LMICs [2,19,61]. In most clinical studies of deliberate self-harm and suicide attempts in Ethiopia, attempters commonly used organophosphate pesticide [22,27,62], which is highly lethal and can be taken impulsively in situations of interpersonal disputes [63]. Death secondary to drowning occurred in our study population in situations where there are deep water wells, with narrow tops, at the backyards of the residential houses in lowland areas and in nearby rivers during the rainy season.

Limitations of this study are that we were restricted to consideration of axis-I disorders during the follow-up. Other factors of potential importance, such as personality disorder, major life-events, stressful life situations and physical illnesses, were not explored. Data presented in this study were combined for all diagnostic categories because of the small number of patients that died from suicide in each group.

## Conclusions

Suicide and suicidal behavior are shown to be common problems in this cohort and severe mental disorders are important risk factors for the behavior. Some of the methods used in this setting are preventable. Our findings indicate that the new government initiative to scale up access to mental health care in Ethiopia also needs to focus on prevention of access to methods of suicide, particularly in people with SMD. Some counter-intuitive findings such as increased risk in married persons and the role of extended family for the subjects in question might need to be examined in further studies and their underlying mechanisms should be explored.

## Abbreviations

SDM: Severe mental disorders; OR: Odds ratio; LMICs: Low income countries; MDD: Major depressive disorder; CIDI 2.1: Composite international diagnostic interview version 2.1; SCAN 2.1: Schedule for clinical interview in neuropsychiatry version 2.1; DSM-IV: Diagnostic statistical manual of mental disorders version IV; ICD-10: International classification of diseases version 10; GAF: Global assessment of functioning; FIS: Family interview schedule; LIFE: Longitudinal interval follow-up evaluation; SPSS: Statistical packages for social sciences; CI: Confidence interval.

## Competing interests

All authors declare that they have no competing interests.

## Authors’ contributions

TS, AA, DK, GM, GK, LJ and AF were responsible for the design of the original study. AF and TS were responsible for designing the LIFE chart tracking of suicidal behavior. TS, GM, CH and AF were primarily responsible for the data analysis. All authors contributed to the write up of the final manuscript and approved the manuscript submission.

## Pre-publication history

The pre-publication history for this paper can be accessed here:

http://www.biomedcentral.com/1471-244X/14/150/prepub

## References

[B1] WHO The Global Burden of Disease: 2004 Update 2008 Geneva: World Health Organization

[B2] Cavanagh JT Carson AJ Sharpe M Lawrie SM Psychological autopsy studies of suicide: a systematic review Psychol Med 2003 33 3 395 405 10.1017/S0033291702006943 12701661

[B3] Hawton K Appleby L Platt S Foster T Cooper J Malmberg A Simkin S The psychological autopsy approach to studying suicide: a review of methodological issues J Affect Disord 1998 50 2–3 269 276 9858086 10.1016/s0165-0327(98)00033-0

[B4] APA Practice guideline for the assessment and treatment of patients with suicidal behaviors Am J Psychiatry 2003 160 Supp. 11 1 6 14649920

[B5] CDC Web-Based Injury Statistics Query and Reporting System (WISQARSTM) 2009 National Center Injury Prevention and Control: Center for Disease Control

[B6] Uwakwe R Gureje O The relationship of comorbidity of mental and substance use disorders with suicidal behaviors in the Nigerian Survey of Mental Health and Wellbeing Soc Psychiatry Psychiatr Epidemiol 2011 46 3 173 180 10.1007/s00127-009-0178-2 20135089

[B7] WHO Suicide Prevention (SUPRE) 2012 Geneva: WHO

[B8] Joe S Stein DJ Seedat S Herman A Williams DR Prevalence and correlates of non-fatal suicidal behaviour among South Africans Br J Psychiatry 2008 192 4 310 311 10.1192/bjp.bp.107.037697 18378997 PMC2701668

[B9] Cassidy F Risk factors of attempted suicide in bipolar disorder Suicide Life Threat Behav 2011 41 1 6 11 10.1111/j.1943-278X.2010.00007.x 21309819

[B10] Posada-Villa J Camacho JC Valenzuela JI Arguello A Cendales JG Fajardo R Prevalence of suicide risk factors and suicide-related outcomes in the National Mental Health Study, Colombia Suicide Life Threat Behav 2009 39 4 408 424 10.1521/suli.2009.39.4.408 19792982

[B11] Sartorius N Jablensky A Korten A Ernberg G Anker M Cooper JE Day R Early manifestations and first-contact incidence of schizophrenia in different cultures. A preliminary report on the initial evaluation phase of the WHO Collaborative Study on determinants of outcome of severe mental disorders Psychol Med 1986 16 4 909 928 10.1017/S0033291700011910 3493497

[B12] Healy D Le Noury J Harris M Butt M Linden S Whitaker C Zou L Roberts AP Mortality in schizophrenia and related psychoses: data from two cohorts, 1875-1924 and 1994-2010 BMJ Open 2012 2 5 10.1136/bmjopen-2012-001810 PMC3488735 23048063

[B13] Balhara YP Verma R Schizophrenia and suicide East Asian Arch Psychiatry 2012 22 3 126 133 23019287

[B14] Koeda A Otsuka K Nakamura H Yambe T Fukumoto K Onuma Y Saga YM Yoshioka Y Mita T Mizugia A Sakai A Endo S Characteristics of suicide attempts in patients diagnosed with schizophrenia in comparison with depression: a study of emergency room visit cases in Japan Schizophr Res 2012 142 1–3 31 39 23127379 10.1016/j.schres.2012.08.029

[B15] Alem A Jacobsson L Kebede D Kullgren G Awareness and attitudes of a rural Ethiopian community toward suicidal behaviour. A key informant study in Butajira, Ethiopia Acta Psychiatr Scand Suppl 1999 397 65 69 10470357 10.1111/j.1600-0447.1999.tb10696.x

[B16] Lester D Suicide and islam Arch Suicide Res 2006 10 1 77 97 10.1080/13811110500318489 16287698

[B17] Bellivier F Yon L Luquiens A Azorin JM Bertsch J Gerard S Reed C Lukasiewicz M Suicidal attempts in bipolar disorder: results from an observational study (EMBLEM) Bipolar Disord 2011 13 4 377 386 10.1111/j.1399-5618.2011.00926.x 21843277

[B18] Bergen H Hawton K Waters K Ness J Cooper J Steeg S Kapur N Premature death after self-harm: a multicentre cohort study Lancet 2012 380 9853 1568 1574 10.1016/S0140-6736(12)61141-6 22995670

[B19] Patel V Ramasundarahettige C Vijayakumar L Thakur JS Gajalakshmi V Gururaj G Suraweera W Jha P Suicide mortality in India: a nationally representative survey Lancet 2012 379 9834 2343 2351 10.1016/S0140-6736(12)60606-0 22726517 PMC4247159

[B20] Niehaus DJ Laurent C Jordaan E Koen L Oosthuizen P Keyter N Muller JE Mbanga NI Deleuze JF Mallet J Stein DJ Emsley R Suicide attempts in an African schizophrenia population: an assessment of demographic risk factors Suicide Life Threat Behav 2004 34 3 320 327 10.1521/suli.34.3.320.42778 15385186

[B21] Adinkrah M Patterns of female suicidal behavior in Ghana Psychol Rep 2011 109 2 649 662 10.2466/09.12.13.17.PR0.109.5.649-662 22238863

[B22] Arap Mengech HN Dhadphale M Attempted suicide (parasuicide) in Nairobi, Kenya Acta Psychiatr Scand 1984 69 5 416 419 10.1111/j.1600-0447.1984.tb02513.x 6730998

[B23] Mekonnen D Kebede Y The prevalence of suicidal ideation and attempts among individuals attending an adult psychiatry out-patient clinic in Gondar, Ethiopia Afr Health Sci 2011 11 1 103 107 21572865 PMC3092311

[B24] Kebede D Alem A Suicide attempts and ideation among adults in Addis Ababa, Ethiopia Acta Psychiatr Scand Suppl 1999 397 35 39 10470353 10.1111/j.1600-0447.1999.tb10692.x

[B25] Kebede D Ketsela T Suicide attempts in Ethiopian adolescents in Addis Abeba high schools Ethiop Med J 1993 31 2 83 90 8513783

[B26] Jacobsson L Suicide and attempted suicide in a general hospital in Western Ethiopia Acta Psychiatrica Scandinevica 1985 71 6 596 600 10.1111/j.1600-0447.1985.tb02553.x

[B27] Bekry AA Trends in suicide, parasuicide and accidental poisoning in Addis Ababa, Ethiopia Ethiop J Health Dev 1999 13 3 247 261

[B28] Kebede D Alem A Shibre T Negash A Fekadu A Fekadu D Deyassa N Jacobsson L Kullgren G Onset and clinical course of schizophrenia in Butajira-Ethiopia–a community-based study Soc Psychiatry Psychiatr Epidemiol 2003 38 11 625 631 10.1007/s00127-003-0678-4 14614550

[B29] Shibre T Spangeus A Henriksson L Negash A Jacobsson L Traditional treatment of mental disorders in rural Ethiopia Ethiop Med J 2008 46 1 87 91 18711994

[B30] Shibre T Kebede D Alem A Negash A Kibreab S Fekadu A Fekadu D Jacobsson L Kullgren G An evaluation of two screening methods to identify cases with schizophrenia and affective disorders in a community survey in rural Ethiopia Int J Soc Psychiatry 2002 48 3 200 208 10.1177/002076402128783244 12413248

[B31] Robins L Wing J Wittchen H Helzer J Babor T Burke J Farmer A Jablenski A Pickens R Regier DA The composite international diagnostic interview. An epidemiologic Instrument suitable for use in conjunction with different diagnostic systems and in different cultures Arch Gen Psychiatry 1988 45 12 1069 1077 10.1001/archpsyc.1988.01800360017003 2848472

[B32] Sartorius N Janca A Psychiatric assessment instruments developed by the World Health Organization Soc Psychiatry Psychiatr Epidemiol 1996 31 2 55 69 10.1007/BF00801901 8881086

[B33] American Psychiatric Association Diagnostic and Statistical Manual of Mental Disorders 1994 IV Washington, DC: American Psychiatric Publishing

[B34] Keller MB Lavori PW Friedman B Nielsen E Endicott J McDonald-Scott P Andreasen NC The longitudinal interval follow-up evaluation. A comprehensive method for assessing outcome in prospective longitudinal studies Arch Gen Psychiatry 1987 44 6 540 548 10.1001/archpsyc.1987.01800180050009 3579500

[B35] Pritchard C Amanullah S An analysis of suicide and undetermined deaths in 17 predominantly Islamic countries contrasted with the UK Psychol Med 2007 37 3 421 430 10.1017/S0033291706009159 17176500

[B36] Christiansen E Larsen KJ Agerbo E Bilenberg N Stenager E Incidence and risk factors for suicide attempts in a general population of young people: a Danish register-based study Aust N Z J Psychiatry 2013 47 3 259 270 10.1177/0004867412463737 23060528

[B37] Choi JW Park S Yi KK Hong JP Suicide mortality of suicide attempt patients discharged from emergency room, nonsuicidal psychiatric patients discharged from emergency room, admitted suicide attempt patients, and admitted nonsuicidal psychiatric patients Suicide Life Threat Behav 2012 42 3 235 243 10.1111/j.1943-278X.2012.00085.x 22380459

[B38] Ruengorn C Sanichwankul K Niwatananun W Mahatnirunkul S Pumpaisalchai W Patumanond J Factors related to suicide attempts among individuals with major depressive disorder Int J Gen Med 2012 5 323 330 22536088 10.2147/IJGM.S30874 PMC3333829

[B39] Chen YW Dilsaver SC Lifetime rates of suicide attempts among subjects with bipolar and unipolar disorders relative to subjects with other Axis I disorders Biol Psychiatry 1996 39 10 896 899 10.1016/0006-3223(95)00295-2 8860192

[B40] Goldstein TR Ha W Axelson DA Goldstein BI Liao F Gill MK Ryan ND Yen S Hunt J Hower H Keller M Strober M Birmaher B Predictors of prospectively examined suicide attempts among youth with bipolar disorder Arch Gen Psychiatry 2012 69 11 1113 1122 22752079 10.1001/archgenpsychiatry.2012.650 PMC3600896

[B41] Fekadu A Alem A Medhin G Shibre T Cleare AJ Prince M Kebede D Utility of the concept of minor depressive disorder: evidence from a large rural community sample in a developing country setting J Affect Disord 2007 104 1–3 111 1118 17448542 10.1016/j.jad.2007.03.008

[B42] Hor K Taylor M Suicide and schizophrenia: a systematic review of rates and risk factors J Psychopharmacol 2010 24 4 Suppl 81 90 10.1177/1359786810385490 20923923 PMC2951591

[B43] Negash A Alem A Kebede D Deyessa N Shibre T Kullgren G Prevalence and clinical characteristics of bipolar I disorder in Butajira, Ethiopia: a community-based study J Affect Disord 2005 87 2–3 193 201 15913783 10.1016/j.jad.2005.03.011

[B44] Siris SG Suicide and schizophrenia J Psychopharmacol 2001 15 2 127 135 10.1177/026988110101500209 11448086

[B45] Suokas JT Perala J Suominen K Saarni S Lonnqvist J Suvisaari JM Epidemiology of suicide attempts among persons with psychotic disorder in the general population Schizophr Res 2010 124 1–3 22 28 20934306 10.1016/j.schres.2010.09.009

[B46] Palmer EJ Connelly R Depression, hopelessness and suicide ideation among vulnerable prisoners Crim Behav Ment Health 2005 15 3 164 170 10.1002/cbm.4 16575794

[B47] Carlborg A Winnerback K Jonsson EG Jokinen J Nordstrom P Suicide in schizophrenia Expert Rev Neurother 2010 10 7 1153 1164 10.1586/ern.10.82 20586695

[B48] Goldsmith SK Pellmar TC Kleinman AM WE WEB Ming T, Tsuang MT, Peter J Reducing Suicide. A National Imperative Textbook of Psychiatric Epidemiology 2011 Third Washington D.C: The national Academic Press 25057611

[B49] Cipriani A Hawton K Stockton S Geddes JR Lithium in the prevention of suicide in mood disorders: updated systematic review and meta-analysis BMJ 2013 346 f3646 10.1136/bmj.f3646 23814104

[B50] Thies-Flechtner K Müller-Oerlinghausen B Seibert W Walther A Greil W Effect of prophylactic treatment on suicide risk in patients with major affective disorders. Data from a randomized prospective trial Pharmcopsychiatry 1996 29 3 103 107 10.1055/s-2007-979553 8738314

[B51] Meltzer HY Alphs L Green AI Altamura AC Anand R Bertoldi A Chouinard G Islam MZ Kane J Krishnan R Lindenmayer JP Pokin S Clozapine treatment for suicidality in schizophrenia: International Suicide Prevention Trial (InterSePT) Arch Gen Psychiatry 2003 60 1 82 91 10.1001/archpsyc.60.1.82 12511175

[B52] Aaron R Joseph A Abraham S Muliyil J George K Prasad J Minz S Abraham VJ Bose A Suicide in young people in rural Southern India Lancet 2004 363 1117 1118 10.1016/S0140-6736(04)15896-0 15064031

[B53] Shah A The relationship between suicide rates and age: an analysis of multinational data from the World Health Organization Int Psychogeriatr 2007 19 6 1141 1152 17433118 10.1017/S1041610207005285

[B54] Phillips MR Yang G Zhang Y Wang L Ji H Zhou M Risk factors for suicide in China: a national case-control psychological autopsy study Lancet 2002 360 1728 1736 10.1016/S0140-6736(02)11681-3 12480425

[B55] Lorant V Kunst AE Huisman M Bopp M Mackenbach J A European comparative study of marital status and socio-economic inequalities in suicide Soc Sci Med 2005 60 11 2431 2441 10.1016/j.socscimed.2004.11.033 15814169

[B56] Masocco M Pompili M Vichi M Vanacore N Lester D Tatarelli R Suicide and marital status in Italy Psychiatry Q 2008 79 4 275 285 10.1007/s11126-008-9072-4 18600458

[B57] Khan MM Mahmud S Karim MS Zaman M Prince M Case–control study of suicide in Karachi, Pakistan Br J Psychiatry 2008 193 402 405 10.1192/bjp.bp.107.042069 18978322

[B58] Devries K Watts C Yoshihama M Kiss L Schraiber LB Deyessa N Heise L Durand J Mbwambo J Jasen H Berhane Y Ellsberg M Garcia-Moreno C Violence against women is strongly associated with suicide attempts: evidence from the WHO multi-country study on women’s health and domestic violence against women Soc Sci Med 2011 73 1 79 86 10.1016/j.socscimed.2011.05.006 21676510

[B59] Oquendo MA Currier D Mann JJ Prospective studies of suicidal behavior in major depressive and bipolar disorders: what is the evidence for predictive risk factors? Acta Psychiatr Scand 2006 114 3 151 158 10.1111/j.1600-0447.2006.00829.x 16889585

[B60] Melle I Barrett EA Insight and suicidal behavior in first-episode schizophrenia Expert Rev Neurother 2012 12 3 353 359 10.1586/ern.11.191 22364334

[B61] Yip PS Caine E Yousuf S Chang SS Wu KC Chen YY Means restriction for suicide prevention Lancet 2012 379 9834 2393 2399 10.1016/S0140-6736(12)60521-2 22726520 PMC6191653

[B62] Abula T Wondmikun Y The pattern of acute poisoning in a teaching hospital, north-west Ethiopia Ethiop Med J 2006 44 2 183 189 17447382

[B63] Yang GH Phillips MR Zhou MG Wang LJ Zhang YP Xu D Understanding the unique characteristics of suicide in China: national psychological autopsy study Biomed Environ Sci 2005 18 6 379 389 16544520

